# Life‐Threatening Acute Hypoxemic Respiratory Failure Caused by Complete Left Lung Collapse Secondary to Mucus Plug Obstruction in a Toddler: A Case Report

**DOI:** 10.1155/crpu/3939387

**Published:** 2026-07-19

**Authors:** Menghak Heng, Chhayhout Chheang, Sophou Chit, Bunleng Kou, Kimhun Ton, Sarin Ean, Chheang Khor, Borin Hem, Vibopha Srey, Sopheak Phy, Heng Leang, Bunpaul Chhar

**Affiliations:** ^1^ Pulmonology Unit, Medical Ward “A”, Calmette Hospital, Phnom Penh, Cambodia; ^2^ Faculty of Medicine, University of Health Sciences, Phnom Penh, Cambodia, akdeniz.edu.tr; ^3^ Outpatient Department, Calmette Hospital, Phnom Penh, Cambodia; ^4^ Pediatric Department, Jayavarman VII Hospital, Siem Reap, Cambodia; ^5^ Anesthesiology Department, Luang Me Hospital, Phnom Penh, Cambodia; ^6^ Anesthesiology Department, Calmette Hospital, Phnom Penh, Cambodia; ^7^ ICU “B” Department, Calmette Hospital, Phnom Penh, Cambodia; ^8^ ICU “A” Department, Calmette Hospital, Phnom Penh, Cambodia; ^9^ Medical Imaging Department, Calmette Hospital, Phnom Penh, Cambodia

**Keywords:** acute hypoxemic respiratory failure, atelectasis, bronchoscopy, lung collapse, mucus plug, pediatric airway obstruction

## Abstract

Acute hypoxemic respiratory failure in toddlers is most commonly associated with severe pneumonia, bronchiolitis, or foreign body aspiration. Complete obstruction of a main bronchus by mucus plugs is an uncommon but potentially life‐threatening cause of unilateral lung collapse and severe hypoxemia. We report the case of a previously healthy 17‐month‐old boy who presented with severe respiratory distress after 3 days of fever and cough. Physical examination revealed marked respiratory distress with absent breath sounds over the left hemithorax. Chest radiography and computed tomography demonstrated complete left lung collapse without evidence of pleural effusion or foreign body. Due to rapidly worsening hypoxemia and suspected central airway obstruction, emergency flexible bronchoscopy was performed under general anesthesia via endotracheal intubation. Complete occlusion of the left main bronchus by thick mucus plugs was identified and successfully removed using suction and bronchoscopic forceps, resulting in immediate improvement in oxygenation and rapid radiologic lung re‐expansion. The patient recovered fully and was discharged in 5 days without complications. This case highlights the importance of considering mucus plug–induced bronchial obstruction in toddlers presenting with sudden unilateral lung collapse and severe hypoxemia, particularly when foreign body aspiration remains a differential diagnosis. Early bronchoscopic intervention can be lifesaving and may prevent prolonged respiratory failure and associated complications.

## 1. Introduction

Acute hypoxemic respiratory failure in young children is a potentially life‐threatening condition commonly caused by severe respiratory infections, bronchiolitis, pneumonia, or foreign body aspiration [1, 2]. In pediatric patients, airway obstruction may rapidly lead to significant hypoxemia because of small airway diameter, reduced pulmonary reserve, and limited ability to clear airway secretions effectively [2, 3].

Mucus is normally produced within the tracheobronchial tree to protect and humidify the respiratory tract. During respiratory infections and inflammatory airway conditions, mucus production may increase substantially and secretions may become thick and tenacious, resulting in mucus plug formation [2]. Although mucus plugging commonly causes partial airway obstruction or segmental atelectasis, complete occlusion of a main bronchus resulting in total unilateral lung collapse is rare, especially in previously healthy toddlers [3–5].

Such presentations may clinically mimic foreign body aspiration, severe pneumonia, or pleural disease, creating diagnostic challenges and delaying definitive management. Prompt recognition and early bronchoscopic intervention are therefore essential to restore airway patency and prevent prolonged hypoxemia and respiratory deterioration.

We report a rare case of acute hypoxemic respiratory failure caused by complete obstruction of the left main bronchus by thick mucus plugs in a previously healthy 17‐month‐old boy.

## 2. Case Presentation

A previously healthy 17‐month‐old boy was referred to a provincial referral hospital with severe respiratory distress and hypoxemia. He had experienced fever, rhinorrhea, and progressive cough for 3 days prior to admission. There was no history of witnessed choking, sudden aspiration event, recurrent respiratory tract infection, prematurity, chronic lung disease, congenital anomaly, or previous hospitalization. Vaccination history was reportedly up to date. There was no known family history of pulmonary disease or smoke exposure.

**Figure 1:**
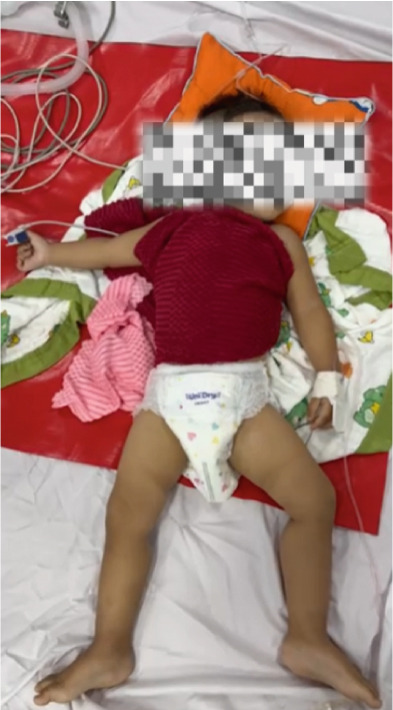
Initial clinical presentation of the 17‐month‐old boy with severe respiratory distress and hypoxemia.

On arrival, the child appeared critically ill with marked respiratory distress. Physical examination demonstrated tachypnea, nasal flaring, intercostal and subcostal retractions, and central cyanosis. Initial oxygen saturation (SpO_2_) was 68% on room air and improved only to 82% despite supplemental oxygen administration via face mask at 10 L/min (Figure 1). Heart rate was 168 beats/min and respiratory rate was 52 breaths/min on presentation. Auscultation revealed markedly diminished air entry over the entire left hemithorax, without wheezing or crackles, whereas breath sounds over the right lung were relatively preserved.

Initial laboratory evaluation demonstrated leukocytosis with a white blood cell count of 16,500/mm^3^, with neutrophil predominance of 78%, and elevated C‐reactive protein of 98 mg/L. Respiratory microbiologic investigations, including viral PCR testing and sputum culture, were not performed because of the emergent nature of the airway intervention and limited local diagnostic availability.

Arterial blood gas analysis while receiving supplemental oxygen demonstrated severe respiratory failure with profound hypoxemia and mild hypercapnia, with pH 7.31, PaO_2_ 48 mmHg, PaCO_2_ 52 mmHg, and HCO_3_^−24^ mmol/L.

**Figure 2:**
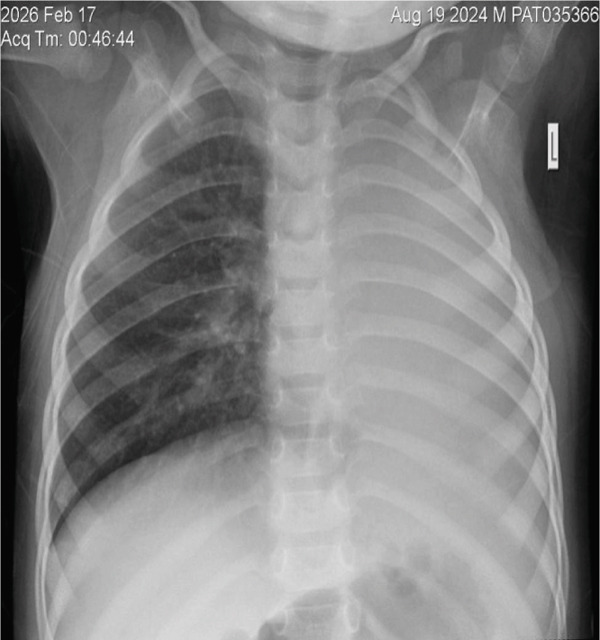
Chest radiograph demonstrating complete left lung collapse with ipsilateral mediastinal shift.

**Figure 3:**
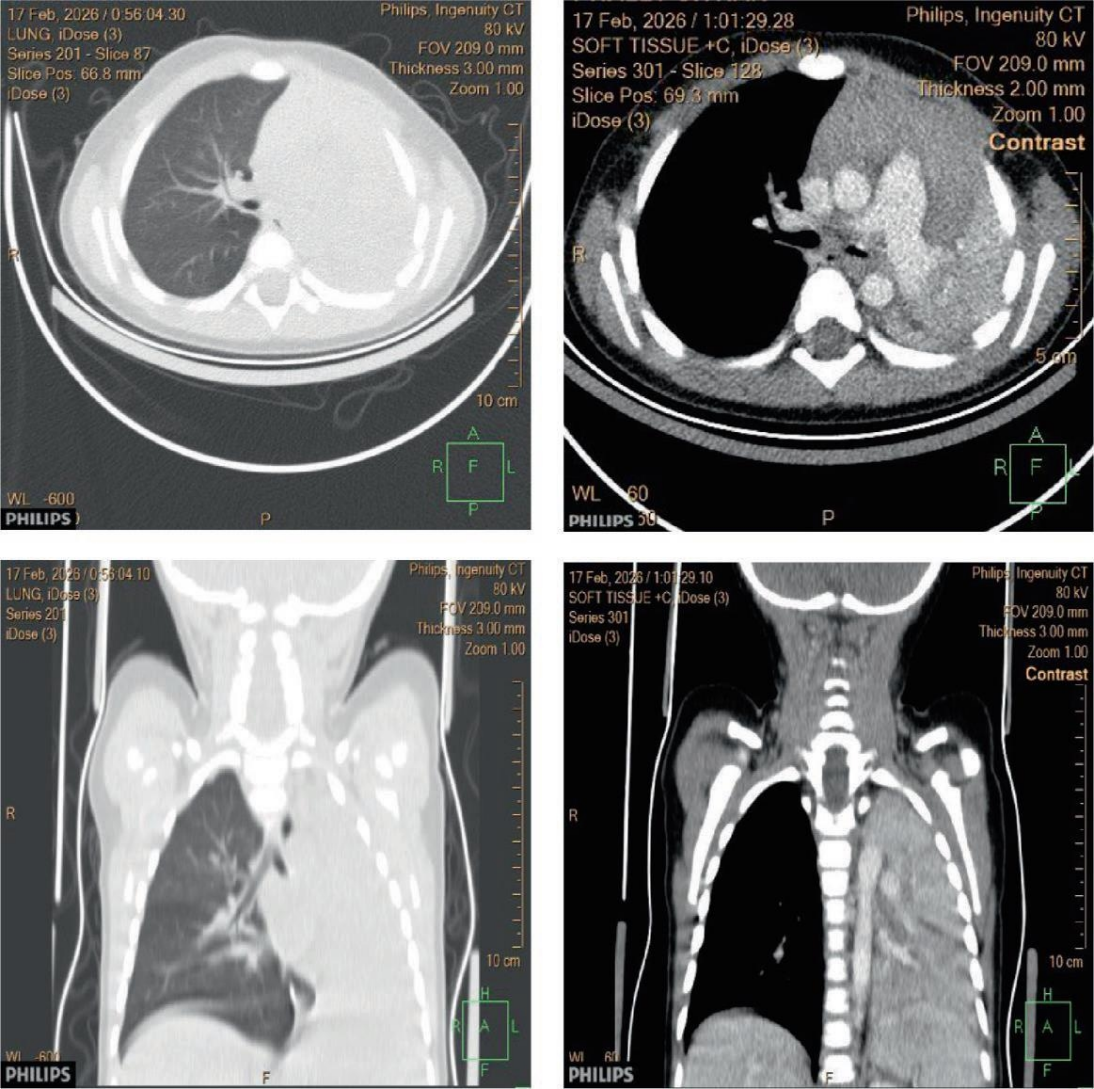
Contrast‐enhanced chest CT scan demonstrating complete left lung atelectasis and obstruction of the left main bronchus without evidence of pleural effusion or foreign body.

Chest radiography demonstrated complete opacification of the left hemithorax with loss of aeration and ipsilateral mediastinal shift, consistent with complete left lung atelectasis (Figure 2). Contrast‐enhanced chest computed tomography confirmed complete collapse of the left lung and obstruction of the left main bronchus without evidence of pleural effusion, mediastinal mass, or radiopaque foreign body (Figure 3).

Given the imaging findings and severe unilateral airway obstruction, differential diagnoses included foreign body aspiration, mucus plug obstruction, and severe endobronchial infection.

Due to rapidly worsening respiratory compromise and suspicion of complete central airway obstruction, emergency flexible bronchoscopy was performed under general anesthesia in the operating room by a multidisciplinary team consisting of pulmonologists, pediatric anesthesiologists, and intensive care physicians. Flexible bronchoscopy was performed using a pediatric flexible bronchoscope (Olympus BF‐XP190, outer diameter 3.1 mm) introduced through a 4.5‐mm internal diameter endotracheal tube via a bronchoscopy adapter while maintaining assisted ventilation throughout the procedure.

**Figure 4:**
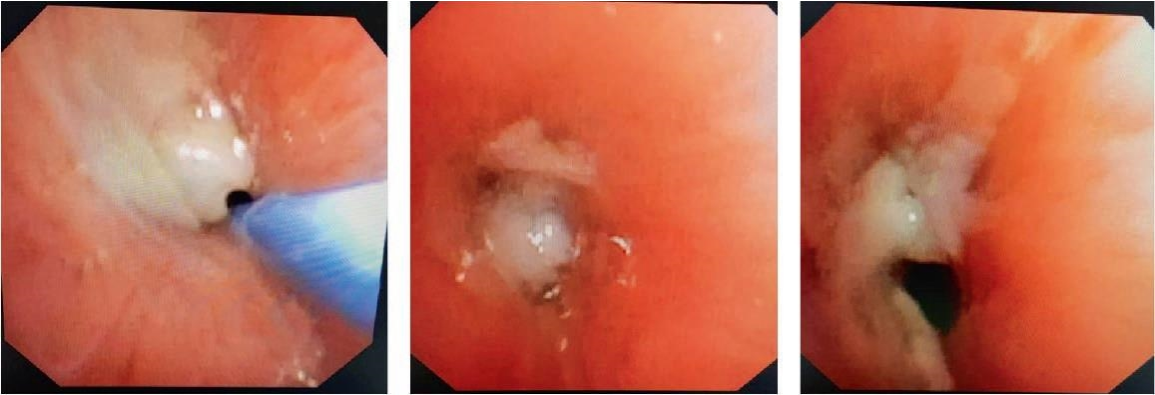
Flexible bronchoscopic view showing complete obstruction of the left main bronchus by thick mucus plugs.

**Figure 5:**
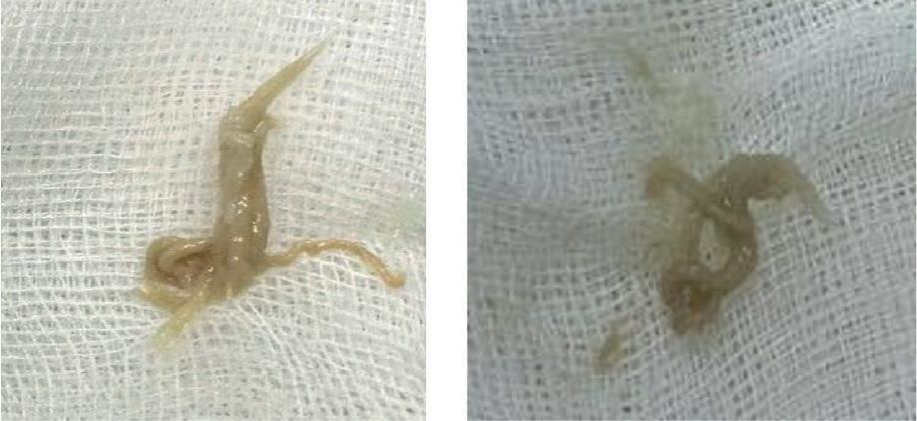
Removal of thick mucus plugs from the left main bronchus using pediatric flexible bronchoscopy.

Bronchoscopic examination revealed complete occlusion of the left main bronchus by thick tenacious mucus plugs (Figure 4). No foreign body or endobronchial structural abnormality was identified. The mucus plugs were successfully removed using suction and bronchoscopic forceps (Figure 5), resulting in immediate restoration of airway patency and rapid improvement in oxygenation.

**Figure 6:**
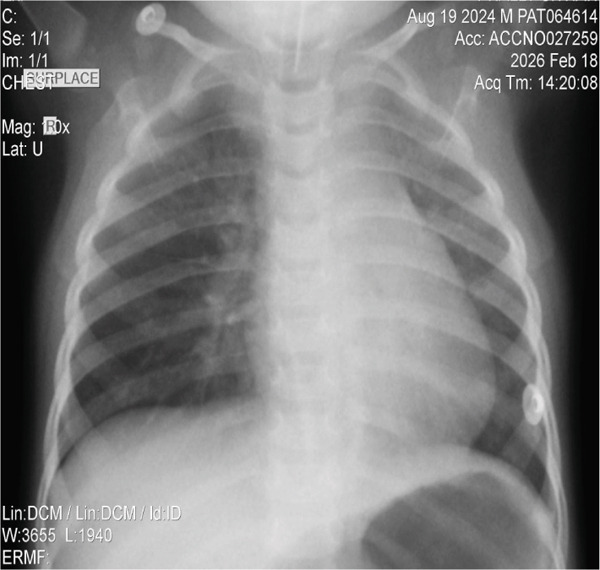
Follow‐up chest radiograph demonstrating re‐expansion of the left lung after bronchoscopic removal of mucus plugs.

Following the procedure, oxygen saturation improved rapidly to 98% on low‐flow supplemental oxygen at 1 L/min, respiratory distress decreased substantially, and repeat chest radiography demonstrated re‐expansion of the left lung (Figure 6). The patient was admitted to the pediatric intensive care unit for close monitoring and supportive care. He continued to improve clinically without further respiratory compromise with SpO_2_ 98% on room air, and was discharged home after 5 days of hospitalization without respiratory or neurologic sequelae.

## 3. Discussion

Complete unilateral lung collapse caused by mucus plug obstruction is uncommon in previously healthy toddlers and may present as a life‐threatening pediatric airway emergency [2, 5]. In young children, narrow airway caliber, immature collateral ventilation, ineffective cough mechanisms, and increased mucus production during respiratory infections predispose to rapid airway obstruction and severe hypoxemia [2, 4].

Foreign body aspiration remains one of the most important differential diagnoses in toddlers presenting with sudden unilateral lung collapse and respiratory distress [3]. In the present case, although there was no witnessed choking episode, foreign body aspiration could not initially be excluded because of the patient′s age, abrupt respiratory deterioration, and imaging findings demonstrating complete unilateral atelectasis. Emergency bronchoscopy therefore served both diagnostic and therapeutic purposes.

Mucus plugs most commonly occur in patients with underlying pulmonary conditions such as asthma, bronchiectasis, cystic fibrosis, chronic infection, or prolonged mechanical ventilation [2, 5]. Complete main bronchus obstruction resulting in total lung collapse in an otherwise healthy child is rarely reported. The severe hypoxemia observed in this patient likely resulted from complete interruption of ventilation to the left lung, leading to significant ventilation‐perfusion mismatch and rapid atelectasis.

Physical examination findings were clinically important in this case. Markedly diminished unilateral breath sounds, severe respiratory distress, and rapid oxygen desaturation prompted urgent imaging evaluation and early suspicion of major airway obstruction. Chest radiography demonstrated complete unilateral opacification with ipsilateral mediastinal shift, findings highly suggestive of obstructive atelectasis rather than pleural effusion. Computed tomography further confirmed complete bronchial obstruction without evidence of mass lesion or foreign body.

Flexible bronchoscopy played a critical role in both diagnosis and management. Bronchoscopic removal of the mucus plug resulted in immediate improvement in oxygenation and rapid lung re‐expansion. Early bronchoscopic intervention is essential in such cases because delayed treatment may result in prolonged hypoxemia, secondary infection, hemodynamic instability, or respiratory arrest [4, 5].

This case also highlights the growing importance of pediatric flexible bronchoscopy capability in Cambodia and other resource‐limited Southeast Asian settings, where access to advanced pediatric airway intervention remains limited outside tertiary referral centers. Prompt multidisciplinary coordination between emergency physicians, pediatricians, pulmonologists, anesthesiologists, and intensive care teams contributed substantially to the favorable clinical outcome in this patient.

This report has several limitations. Some physiologic and laboratory parameters were incompletely documented retrospectively. In addition, microbiologic investigations were limited because of the emergent clinical setting and local resource availability. Nevertheless, this case provides important bronchoscopic and radiologic documentation of complete unilateral lung collapse caused by mucus plug obstruction in a previously healthy toddler.

## 4. Conclusion

Complete obstruction of a main bronchus by mucus plugs is a rare but potentially life‐threatening cause of acute hypoxemic respiratory failure and total lung collapse in toddlers. Clinicians should maintain a high index of suspicion for central airway obstruction in children presenting with severe respiratory distress, unilateral absent breath sounds, and sudden unilateral lung collapse, particularly when foreign body aspiration remains a differential diagnosis. Prompt imaging evaluation and early bronchoscopic intervention are essential for diagnosis and can be lifesaving.

## Author Contributions

Menghak Heng was responsible for patient management, conception of the case report, data collection, bronchoscopic interpretation, and drafting of the initial manuscript. Chhayhout Chheang, Sophou Chit, and Bunleng Kou contributed to clinical interpretation, literature review, and critical revision of the manuscript. Kimhun Ton and Heng Leang contributed to imaging interpretation and diagnostic evaluation. Sarin Ean, Chheang Khor, Borin Hem, Vibopha Srey, and Sopheak Phy contributed to anesthetic airway management during bronchoscopy and critically reviewed the manuscript. Bunpaul Chhar supervised the overall work, provided senior clinical oversight, critically revised the manuscript for important intellectual content, and approved the final version for submission.

## Funding

No funding was received for this manuscript.

## Disclosure

All authors read and approved the final manuscript and agree to be accountable for all aspects of the work in accordance with ICMJE authorship criteria.

## Ethics Statement

Written informed consent was obtained from the patient′s parents for publication of this case report and accompanying images.

## Conflicts of Interest

The authors declare no conflicts of interest.

## Data Availability

The data that support the findings of this study are available on request from the corresponding author. The data are not publicly available due to privacy or ethical restrictions.
